# Tile-based microscopic image processing for malaria screening using a deep learning approach

**DOI:** 10.1186/s12880-023-00993-9

**Published:** 2023-03-22

**Authors:** Fetulhak Abdurahman Shewajo, Kinde Anlay Fante

**Affiliations:** Faculty of Electrical and Computer Engineering, Jimma University, 378 Jimma, Ethiopia

**Keywords:** Deep learning, Malaria, Object detection, *Plasmodium falciparum*, Thick smear microscopic image, Tile-based image processing, YOLOV4

## Abstract

**Background:**

Manual microscopic examination remains the golden standard for malaria diagnosis. But it is laborious, and pathologists with experience are needed for accurate diagnosis. The need for computer-aided diagnosis methods is driven by the enormous workload and difficulties associated with manual microscopy based examination. While the importance of computer-aided diagnosis is increasing at an enormous pace, fostered by the advancement of deep learning algorithms, there are still challenges in detecting small objects such as malaria parasites in microscopic images of blood films. The state-of-the-art (SOTA) deep learning-based object detection models are inefficient in detecting small objects accurately because they are underrepresented on benchmark datasets. The performance of these models is affected by the loss of detailed spatial information due to in-network feature map downscaling. This is due to the fact that the SOTA models cannot directly process high-resolution images due to their low-resolution network input layer.

**Methods:**

In this study, an efficient and robust tile-based image processing method is proposed to enhance the performance of malaria parasites detection SOTA models. Three variants of YOLOV4-based object detectors are adopted considering their detection accuracy and speed. These models were trained using tiles generated from 1780 high-resolution *P. falciparum*-infected thick smear microscopic images. The tiling of high-resolution images improves the performance of the object detection models. The detection accuracy and the generalization capability of these models have been evaluated using three datasets acquired from different regions.

**Results:**

The best-performing model using the proposed tile-based approach outperforms the baseline method significantly (Recall, [95.3%] vs [57%] and Average Precision, [87.1%] vs [76%]). Furthermore, the proposed method has outperformed the existing approaches that used different machine learning techniques evaluated on similar datasets.

**Conclusions:**

The experimental results show that the proposed method significantly improves *P. falciparum* detection from thick smear microscopic images while maintaining real-time detection speed. Furthermore, the proposed method has the potential to assist and reduce the workload of laboratory technicians in malaria-endemic remote areas of developing countries where there is a critical skill gap and a shortage of experts.

## Background

Malaria is one of the most fatal diseases and the cause of high mortality rate in the developing countries, which is transmitted from infected to healthy humans through the bites of female Anopheles mosquitoes. It is caused by a unicellular parasite known as plasmodium. Once inside the human body, the parasite grows inside the liver before being released into the bloodstream to infect red blood cells (RBCs). Plasmodium parasites are classified into five species: *P. falciparum* (*P. falciparum*), *P. vivax* (*P. vivax*), *P. ovale* (*P. ovale*), *P. Knowlesi* (*P. knowlesi*), and *P. malariae* (*P. malariae*). *P. falciparum* and *P. vivax* are the most pathogenic and constitutes the majority of the malaria cases. According to the World Health Organization (WHO) malaria report, an estimated 241 million malaria cases were reported in 2021, with 95% of cases occurring in Africa and only 5% occurring outside of Africa. There were 627,000 malaria deaths globally in 2021, among which 96% of the cases occurred in 29 countries. Six countries, Nigeria (27%), the Democratic Republic of Congo (12%), Uganda (5%), Mozambique (4%), Angola (3.4%) and Burkina Faso (3.4%), accounted for about 55% of all cases worldwide [1].

Malaria diagnosis can be accomplished through a variety of methods, including clinical diagnosis, microscopic diagnosis, rapid diagnostic test kits (RDTs), and polymerise chain reaction (PCR). Clinical diagnosis uses various malaria symptoms such as fever history. It has low specificity, leading to significant antimalarial drug overuse [2]. PCR is the most sensitive method but costly and complex, whereas RDTs are highly sensitive and unable to quantify parasite density [3]. Parasitological confirmation by microscopy using thin/thick blood film remains the golden standard for malaria diagnosis, but it is a cumbersome method [4]. In the procedures of manual microscopy diagnosis, a thin or thick blood smear is prepared by spreading a drop of blood on a glass slide which is dried and stained before being visually examined by a microscopist for parasite identification. A thick smear has a large volume of blood and a large number of parasites per blood volume. It is usually used to determine whether the patient’s blood contains malaria parasites. A thin blood smear has low blood volume and less number of parasites per blood volume, and it is often used both for parasitemia detection and identification of parasite species and their life stage.

The accuracy of manual microscopic examination is severely hampered by high intra/inter-observer variability, which is exacerbated by the large number of cases diagnosed per day in malaria-endemic areas with limited resources [5]. Besides, visual examination using manual microscopy is tedious and time-consuming [6]. The shortage of trained experts and lack of a rigorous system to support expertise skill gaps in malaria- endemic regions lead to incorrect diagnosis results which contribute to inappropriate treatment [7, 8]. The challenges in the manual microscopy diagnosis procedure have motivated the research community to develop automated computer-aided diagnostic (CAD) systems to improve malaria diagnosis accuracy and reduce the clinical challenges due to human error by empowering microscopists’ diagnostic decisions for improved patient treatment.

To develop automated malaria diagnosis systems, most existing studies have combined combine traditional image processing techniques with classical machine learning algorithms. Traditional image processing methods, such as adaptive threshold techniques and morphological operations, have been used to separate the parasite candidates from the background of either thick or thin smear microscopic images [8–11]. However, because the parameters of segmentation techniques are determined empirically, such image processing approaches are highly sensitive to variations in image quality. Conventional machine learning models, on the other hand, use handcrafted features from segmented malaria parasite regions of interest (ROI) to classify malaria-infected and uninfected cells [11–13]. However, classical machine learning algorithms, which are based on hand-engineered features, struggle to generalize as the quality variation of their input data increases. Deep learning algorithms have recently made significant advances in their application to a variety of medical imaging tasks, including image segmentation and reconstruction [14, 15], classification tasks [16–18], and object detection [19]. Furthermore, recent studies show that deep learning-based algorithms outperform conventional image processing and machine learning techniques for detecting and identifying malaria parasites from microscopic images of thin and thick blood smears [20, 21].

The state-of-the-art (SOTA) deep learning-based object detection models were trained and evaluated on large-scale datasets such as ImageNet [22], Pascal VOC [23], and MS COCO [24], which are generic and largely represent medium and large-scale objects. Although the SOTA deep learning-based object detection models perform well for medium and large-scale object detection tasks, their direct application for small object detection tasks, such as malaria parasites in microscopic images of blood films, do not achieve a satisfactory performance [25, 26]. When an input image is processed through the various layers of SOTA object detection models, it loses a significant amount of spatial information, which is critical for small object localization. Especially, when the input image is high resolution (HR), it must be resized to fit within the detection network’s input size. This is because existing object detection models use relatively low-resolution network input sizes to keep computational demands low. While it is useful to use a high-resolution camera for the identification of small objects, processing HR images using SOTA deep learning models requires compressing the high-resolution image to a low-resolution network input size. This leads to massive spatial information loss, which exacerbates the small object detection problem for SOTA deep learning-based object detection models.

Pathologists can now acquire HR images, which are useful for a variety of clinical management tasks, due to advancements in digital data acquisition technologies in medical imaging [27]. Deep learning algorithms, on the other hand, face a formidable computational challenge when dealing with HR images. Similarly, microscopic images captured through a microscope’s eyepiece with a digital camera or smartphone camera have a high resolution [7, 28]. The previous studies for malaria parasites detection [20, 29] have fed HR images to SOTA deep learning-based object detectors by simply downsampling the image. However, feeding downsampled HR microscopic images to SOTA deep learning models to detect very small objects, such as *P. Falciparum*, is nearly impossible. The goal of this study is to investigate an effective and robust method for processing HR thick smear microscopic images in computationally efficient SOTA deep learning-based object detection algorithms without degrading the detailed information found in the original HR images. In this study, tile-based image processing is proposed to improve the small object detection accuracy of computationally efficient SOTA deep learning-based object detection models, which are limited in their ability to process HR images due to their network input resolution. Despite the fact that there are similar research works that investigate tile-based image processing for other problem domains such as remote sensing imagery [30, 31], to the best of our knowledge, no other work has used tile-based microscopic image processing for malaria parasite screening. Because the parasites are sparsely distributed throughout the microscopic image, the proposed method does not use dynamic tile processing. The proposed method has significantly improved the performance of SOTA object detection models while incurring little performance degradation in the models’ inference speed.

The contributions of this study are listed below.

1. An effective and robust tile-based high-resolution thick smear microscopic image processing approach is employed to detect *P. falciparum* using SOTA deep learning-based object detection models. The proposed method significantly improves the performance of SOTA *P. falciparum* detection models with minimal effect on inference speed.
2. Extensive experimental analysis with varying configurations and evaluation strategies was carried out to investigate the detection performance and robustness of SOTA object detection models using datasets collected from various regions.
3. A thorough comparison of the performance of the proposed method with baseline and previously reported results was carried out. On three representative datasets for *P. falciparum* detection, the proposed method outperformed baseline methods and previously proposed models.

## Related works

Several attempts have been made over the last decade to develop efficient automated malaria parasite detection models from thin and thick blood smear microscopic images. However, the majority of such automated diagnosis tools were created using traditional image processing techniques and traditional machine learning approaches based on handcrafted features. For in-depth discussions on these topics, readers should consult the following literature: [3, 32–36].

Recently, advances in the field of machine learning with convolutional neural networks (CNNs) have piqued the interest of researchers for medical image analysis, including the identification of malaria parasites from thin and thick blood smear microscopic images [37]. This is due to their superior performance compared to hand-crafted feature extraction-based techniques by automatically learning robust feature representation from raw image pixel values [38]. Several studies, as described below, demonstrated the applicability of deep learning algorithms for the identification of malaria parasites from microscopic images of both thin and thick blood film.

The authors of [7] proposed a two-stage malaria parasite detection model that included parasite candidate selection using the intensity value of grayscale thick blood film microscopic images and CNN-based classification. Unfortunately, the proposed parasite candidate selection technique is ineffective because traditional image processing techniques are inefficient when applied to images obtained under different environmental conditions. Furthermore, the candidate parasite selection method has a direct impact on the CNN classifier’s performance. Another study [20] proposed a multi-pipeline approach in which Mask-RCNN was used as a pre-candidate *P. falciparum* and *P. vivax* species detector, followed by a classifier head to filter out false positives. The authors used experimentally defined threshold scores to evaluate their detection system at the image and patient levels. They reported image-level accuracy of 90.8% and patient-level accuracy of 97.6%. However, no patch level evaluation is provided. A dual deep learning framework for red blood cell (RBC) segmentation from thin smear microscopic images was reported in [21]. A U-Net model was used as a pre-candidate RBC cluster segmentation method, and Faster-RCNN was used for final RBC detection. This study, however, does not distinguish between malaria-infected and uninfected RBCs.

A mobile-based *P. falciparum* and white blood cell (WBC) localization using pre-trained deep learning models were proposed in [29]. In this study, the authors created a new dataset of 903 fields stained with thick blood smear microscopic images to train and evaluate their models. Another study in [39] proposed an ensemble of pre-trained and custom CNN models for the classification of infected and uninfected RBC cells segmented from thin blood smear microscopic images. Modified versions of the YoloV3 and YoloV4 models were proposed in [40, 41] to improve the detection capability of these models from thick smear microscopic images and to make them lightweight enough to be integrated with mobile phone-based diagnosis applications.

Due to the small size of the malaria parasite, detecting it from thick smear microscopic images is extremely difficult. Most existing deep learning models perform poorly in detecting these parasites in thick smear images. However, thick blood film is the most commonly used slide preparation technique for malaria diagnosis, and the development of robust automated tools is critical in reducing the difficulties associated with manual microscopy based malaria diagnosis. The majority of existing studies on malaria parasite detection or classification use thin smear blood films [42–46]. This could be due to the ease with which infected and uninfected RBCs can be distinguished due to their larger size in thin film blood smear microscopic images.

## Materials and methods

**Fig. 1:**
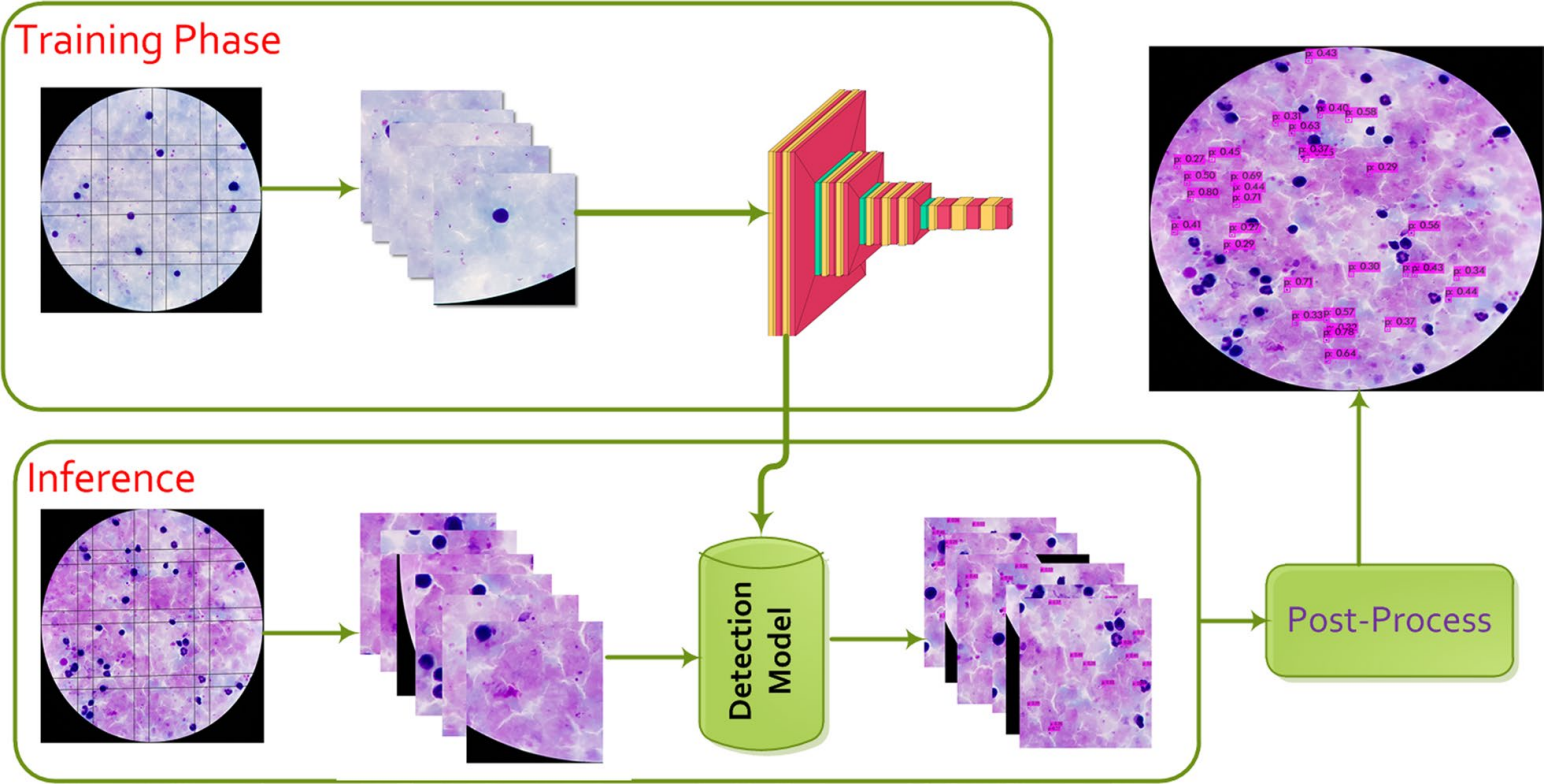
General overview of the proposed framework. First, the high-resolution image is sliced into overlapping tiles and fed as an input to the detection network for training. Then, the trained model is used during inference to predict parasite location at individual tiles. Finally, the initial prediction results are fused to generate refined detections to be superimposed on the input high-resolution image

This study integrates tile-based image processing with object detection models to improve the detection performance of SOTA deep learning-based *P. falciparum* detection from thick smear microscopic HR images. The tile-based image processing is introduced to increase the small object detection capability of SOTA object detection algorithms from high-resolution thick smear microscopic images than their network input resolution allows. The general overview of the proposed scheme is depicted in Fig. 1.

The proposed method divides the HR image into overlapping small images called tiles, which can then be fed into object detection networks without downsampling the original image resolution. During model training, tiles containing the object of interest (*P. falciparum*) were fed into the model, while tiles containing no objects of interest were excluded. Some objects may be cut at tile boundaries during the tiling process, and the ratio of areas between partially included parts in the tile and the complete object was used to decide whether to keep or discard the annotation during model training. Dividing HR images into tiles increases the relative area of objects in an image that can be fed to object detection models.

Similarly, at inference time, the high-resolution image was divided into smaller overlapping tiles, and initial detection results were obtained for individual tiles. The initial tile-level detection results were then merged, and the non-maximum suppression (NMS) algorithm was used to avoid duplicate detections at overlapping regions with a 30% intersection over union (IOU) threshold. Finally, the refined detection results were then stitched onto the high-resolution input images. The proposed tile-based approach demonstrated that computationally efficient SOTA deep learning models, which are limited by their network input resolution to process HR images, detect small objects with increased detection accuracy while without significantly increasing computational overhead during inference time.

### Datasets

**Fig. 2:**
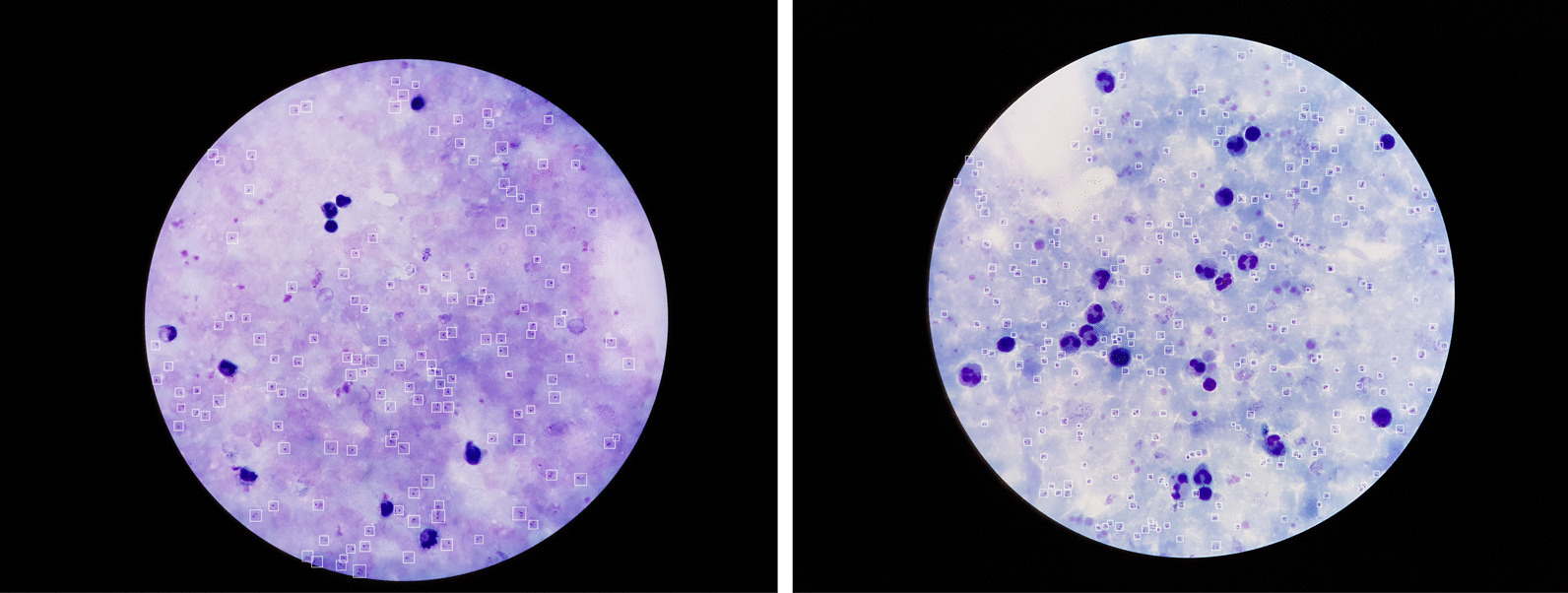
Sample images taken from the model development dataset. The boxes indicate ground truth bounding box locations of *P. falciparum*

Experiments were conducted with two types of datasets to validate the effectiveness of the proposed approach in this work. The first, known as the model development dataset, is used for training, validation, and testing the proposed SOTA detection models before selecting the best model based on detection accuracy and computation speed. This dataset consists of high-resolution thick smear microscopic images with *P. falciparum* which is acquired by researchers in Bangladesh [7]. It is collected from 150 patients at Chittagong Medical College Hospital, Bangladesh, and manually annotated by experienced experts. There are an average of 12 images per patient and 47 parasites per image in the dataset as listed in Table 1. The images have a high resolution of 4032 $\times$ 3024 pixels and are in RGB color format. Figure 2 depicts examples of images from the development dataset with their corresponding annotations.

**Table 1:** Description of publicly available datasets used in this study

	Training	Validation	Testing	Total
*Model development dataset* [7]				
Number of patients	96	24	30	150
Number of images	1140	266	374	1780
Number of parasites	49,520	11,174	22,783	83,477
*External dataset 1* [10]				
Number of images	958	106	118	1182
Number of parasites	6214	694	719	7628
*External dataset 2* [29]				
Number of images	664	166	100	930
Number of parasites	7734	470	1035	9239
*External dataset 3* [20]				
Number of images	–	–	–	1141

The second dataset, collected from various regions, is used as an external dataset to further evaluate the proposed model’s generalisation capability, which was chosen based on its performance on the model development dataset. Furthermore, the external dataset was combined with the model development dataset to fine-tune the chosen model. The external dataset is composed of three distinct datasets. The first external dataset consists of a low-resolution (750 $\times$ 750 pixels ) thick smear microscopic images with *P. falciparum* collected from 133 individuals [10]. The second external dataset is also a collection of thick smear microscopic images obtained from Mulago National referral hospital in Uganda [29]. This dataset contains 903 high-resolution images with dimensions of 3264 $\times$ 2448 pixels. The third external dataset is used to test performance of the proposed detection model on negative images. This dataset consists of 1141 thick smear microscopic images obtained from 50 uninfected patients [20]. The datasets are all publicly available and captured by attaching a mobile phone camera to the microscope’s eyepiece, and they differ in staining style and imaging characteristics. A comprehensive descriptions of the datasets is given Table 1.

### The proposed malarial parasites detection networks

The proposed *P. falciparum* detection algorithms use the YOLOV4 object detection model [47], which was chosen due to its high detection performance and inference speed compared to other single-stage and two-stage detectors [47]. Three different YOLOV4-based object detection models were evaluated in this study to find the best model with the best trade-off between detection performance and inference speed. The first detection network, dubbed YOLOV4-MOD, is based on our previous work [41] and it has been modified to improve small object detection performance of the original YOLOV4 model while requiring minimal computation. This model has many convolution layers and a large number of trainable parameters. The other two detection networks are based on lightweight YOLOV4 models known as tiny YOLOV4 models [48], which are designed for faster inference while sacrificing detection accuracy. The first of these lightweight models, called YOLOV4-tiny, has two detection heads, while the second, called YOLOV4-tiny-3 l, has three detection heads. Increasing the number of detection heads of YOLO-based models improves their detection performance for small objects [41]. In comparison to the large-size model, these two lightweight networks have fewer trainable parameters and fewer convolution and max-pooling layers.

### Evaluation metrics

In this study, two commonly used evaluation metrics for object detection tasks, namely *average precision (AP) * and *recall (R)*, were used to evaluate performance of the proposed models. The average precision is computed from the area under interpolated Precision-Recall Curve (PRC), whereas precision is calculated as the ratio of the number of true positive detections to all detected objects. The recall measures the fraction of detections that are true positive. The evaluation metric formulas are provided below.

1 $$\begin{aligned} Precsion=\; & {} \frac{|TP|}{|TP + FP|} \end{aligned}$$

2 $$\begin{aligned} Recall=\; & {} \frac{|TP|}{|TP + FN|} \end{aligned}$$

3 $$\begin{aligned} AP=\; & {} \frac{1}{11} \sum _{r\epsilon \{0.0,...1.0\}} p_{interp}(r) \quad \end{aligned}$$

where

$$\begin{aligned} p_{interp}(r) = \max _{{\widetilde{r}} \ge r} p({\widetilde{r}}) \end{aligned}$$

where True Positive (TP) denotes the number of correctly detected objects, False Positive (FP) denotes the number of incorrectly predicted suspicious objects, and False Negative (FN) denotes the number of undetected objects. $p_{interp}$ represents the interpolated precision (p) over a given recall (r) values in an ascending order from 0.0 to 1.0 into 11 points - 0, 0.1, 0.2,..., 0.9 and 1.0.

## Results

### Experimental setup

Several experimental settings were used in this study to assess the efficacy of the proposed tile-based thick smear microscopic image processing approach for malaria parasite screening. First, the three proposed YOLOV4 based object detection models (YOLOV4-MOD, YOLOV4-tiny, and YOLOV4-tiny-3 L) were trained using tiles with different sizes generated from the model development dataset described in Table 1. Afterwards, the best model is selected considering the trade-off between computation speed and detection performance on the model development test data. The dataset is divided into training, validation, and testing at patient level where images from 96 patients were used for training, images from 24 patients for validation, and images from 30 patients for testing. In this experimental setting, optimal network hyper-parameters of the detection models were also selected by using the model development validation dataset.

After the selection of the best model among the three proposed detection models, its *P. falciparum* detection performance was tested on the external dataset 1 and external dataset 2 (see Table 1). When the selected model was evaluated using external dataset 1, tile-based processing was not applied since the resolution of the images in the dataset was not high compared to the proposed detection network’s input resolution. However, external dataset 2 consists of high-resolution thick smear microscopic images, and the tile-based approach was applied when the performance evaluation of the models were carried out. Furthermore, using these two external datasets, two experiments were set up during the evaluation phase of the selected model. The selected model was evaluated in the first experimental setup by using the entire dataset as test data. The datasets were partitioned into training, validation, and test in the second experimental setting, as shown in Table 1, and combined with the model development dataset to fine-tune the selected model.

In addition, the selected model was evaluated on 1141 images without malarial parasites collected from 50 uninfected people [20], which have not been used during model training. This dataset consists of a high-resolution thick smear microscopic images with a resolution of 4032 $\times$ 3024 pixels. This experiment was carried out to assess the performance of the selected model in terms of specificity.

Finally, the proposed tile-based approach was compared with a baseline method in which a full high-resolution image is downscaled to the proposed SOTA detection network’s input resolution during model training and inference phases. This baseline model outperforms the proposed tile-based approach in terms of training and inference speed. However, downscaling the images results in significant information loss, resulting in a massive degradation of detection accuracy.

**Table 2:** Comparisons of detection performance and inference speed for YOLOV4-MOD by using the proposed and baseline approach on model development test set data

Model	Tile size		AP (%)	R (%)	Time (sec/img)
	Train	Inference			
YOLOV4-MOD@416	1088 $\times$ 1088	1088 $\times$ 1088	83.1	94.3	3.0
		832 $\times$ 832	**85.5**	**95.1**	4.0
		608 $\times$ 608	82.8	91.3	8.0
YOLOV4-MOD@512	1088 $\times$ 1088	1088 $\times$ 1088	81.8	90.5	4.0
		832 $\times$ 832	82.5	92.6	5.0
		608 $\times$ 608	78.2	91.7	9.0
YOLOV4-MOD@416	832 $\times$ 832	1088 $\times$ 1088	80.6	90.8	3.0
		832 $\times$ 832	85.0	93.5	4.0
		608 $\times$ 608	75.0	86.1	7.0
YOLOV4-MOD@512	832 $\times$ 832	1088 $\times$ 1088	66.1	82.3	4.0
		832 $\times$ 832	82.5	93.2	5.0
		608 $\times$ 608	79.6	92.7	10.0
YOLOV4-MOD@416	608 $\times$ 608	1088 $\times$ 1088	43.5	63.3	3.0
		832 $\times$ 832	66.7	82.5	3.5
		608 $\times$ 608	76.8	90.6	6.0
YOLOV4-MOD@512	608 $\times$ 608	1088 $\times$ 1088	55.3	85.7	3.0
		832 $\times$ 832	69.0	85.6	5.0
		608 $\times$ 608	77.9	90.1	8.5
*Without tiling*					
YoloV4-MOD@512	–	–	79.78	80	0.25
YoloV4-MOD@416	–	–	72.8	76	0.2

Bold values indicate the best-performing model

### Proposed detection network training and hyper-parameter optimization

A publicly available high-resolution thick smear microscopic image dataset [7] was used for proposed model training, hyperparameter optimization, and model selection. To prevent the loss of detailed information in HR images and to keep the computation cost optimal, different tile sizes relative to the detection network’s input resolution were used. To address the issue of a limited dataset in all experimental settings, pre-trained models using the MS COCO dataset [24] were used, and fine-tuning was performed using the target dataset.

The default configurations of the original versions of the selected YOLOV4-based models were used in this work, unless otherwise specified. Anchor box sizes were adjusted based on the network input resolution and ground truth bounding box information of the specific dataset used for training. A batch size of 2 was used for large YOLOV4-based models (YOLOV4-MOD) and batch size of 8 was used for lightweight models (YOLOV4-tiny and YOLOV4-tiny-3 l). All models were trained for 4000 iterations with the default settings for data augmentation, optimizer, and loss functions. The initial learning rate for the large-size model was 0.001 and 0.00261 for the lightweight models, and it was decreased by a factor of 10 at 80% and 90% of the training iteration, respectively. Due to computational constraints, the detection network input sizes were set as 416 $\times$ 416 and 512 $\times$ 512 for YOLOV4-MOD model, and 416 $\times$ 416, 512 $\times$ 512, and 608 $\times$ 608 for the two lightweight models. All experiments were carried out using Google Colaboratory and an NVIDIA TESLA K80 processor with 12 GB of RAM.

**Table 3:** Comparisons of detection performance and inference speed for YOLOV4-tiny by using the proposed and baseline approach on model development test set data

Model	Tile size		AP (%)	R (%)	Time (sec/img)
	Train	Inference			
YOLOV4-tiny@416	1088 $\times$ 1088	1088 $\times$ 1088	56.0	60.0	1.0
		832 $\times$ 832	67.0	73.0	1.0
		608 $\times$ 608	78.0	86.0	1.4
		416 $\times$ 416	65.0	75.8	1.4
YOLOV4-tiny@512	1088 $\times$ 1088	1088 $\times$ 1088	70.0	75.9	1.0
		832 $\times$ 832	80.4	86.1	1.2
		608 $\times$ 608	82.6	91.4	1.6
YOLOV4-tiny@608	1088 $\times$ 1088	1088 $\times$ 1088	77.9	83.4	1.0
		832 $\times$ 832	85.9	92.7	1.3
		608 $\times$ 608	81.9	89.3	1.7
YOLOV4-tiny@416	832 $\times$ 832	1088 $\times$ 1088	53.3	57.6	1.0
		832 $\times$ 832	66.0	71.9	1.1
		608 $\times$ 608	81.4	88.1	1.6
YOLOV4-tiny@512	832 $\times$ 832	1088 $\times$ 1088	66.7	72.8	1.0
		832 $\times$ 832	79.1	85.0	1.1
		608 $\times$ 608	86.0	94.1	1.6
		416 $\times$ 416	78.8	89.2	2.7
YOLOV4-tiny@608	832 $\times$ 832	1088 $\times$ 1088	76.7	82.6	1.0
		832 $\times$ 832	86.0	92.9	1.3
		608 $\times$ 608	84.4	91.5	1.6
YOLOV4-tiny@416	608 $\times$ 608	1088 $\times$ 1088	48.0	57.1	1.0
		832 $\times$ 832	60.9	67.1	1.0
		608 $\times$ 608	83.2	90.1	1.3
		416 $\times$ 416	84.0	91.1	2.0
YOLOV4-tiny@512	608 $\times$ 608	1088 $\times$ 1088	54.3	60.8	1.0
		832 $\times$ 832	76.4	79.8	1.0
		608 $\times$ 608	**87.1**	**95.3**	1.5
		416 $\times$ 416	84.0	94.9	2.6
YOLOV4-tiny@608	608 $\times$ 608	1088 $\times$ 1088	69.4	85.7	1.0
		832 $\times$ 832	84.3	92.1	1.3
		608 $\times$ 608	87.0	95.3	1.9
*Without tiling*					
YoloV4-tiny@416	–	–	54.0	21.0	0.15
YoloV4-tiny@512	–	–	69.0	48.0	0.15
YoloV4-tiny@608	–	–	76.0	57.0	0.15

Bold values indicate the best-performing model

The proposed tile-based image processing method introduces new parameters, such as tile size and overlapping ratio, that must be tuned during model validation. The tile sizes were chosen based on the input size of the proposed detection networks. In comparison to the detection networks’ input resolution, tile sizes that were too large or too small were not chosen. Selecting large tile sizes contribute to the loss of detailed information due to downsampling of the tiles to fit onto the network input. On the other hand, choosing too small tile sizes increases computation time due to the large number of tiles generated per image and necessitates upsampling the tiles to fit onto the network input size.

**Table 4:** Comparisons of detection performance and inference speed for YOLOV4-tiny-3 l by using the proposed and baseline approach on model development test set data

Model	Tile size		AP (%)	R (%)	Time (sec/img)
	Train	Inference			
YOLOV4-tiny-3l@416	1088 $\times$ 1088	1088 $\times$ 1088	55.4	59.8	1.0
		832 $\times$ 832	65.9	71.2	1.0
		608 $\times$ 608	76.9	84.6	1.4
YOLOV4-tiny-3l@512	1088 $\times$ 1088	1088 $\times$ 1088	67.2	72.8	1.3
		832 $\times$ 832	78.0	84.3	1.4
		608 $\times$ 608	83.4	92.3	2.0
YOLOV4-tiny-3l@608	1088 $\times$ 1088	1088 $\times$ 1088	78.4	84.3	1.2
		832 $\times$ 832	86.1	93.0	1.6
		608 $\times$ 608	81.6	89.3	2.0
YOLOV4-tiny-3l@416	832 $\times$ 832	1088 $\times$ 1088	50.0	53.7	1.0
		832 $\times$ 832	64.3	70.0	1.0
		608 $\times$ 608	80.5	87.7	1.5
YOLOV4-tiny-3l@512	832 $\times$ 832	1088 $\times$ 1088	64.9	71.4	1.1
		832 $\times$ 832	78.1	84.5	1.4
		608 $\times$ 608	85.7	94.2	1.9
YOLOV4-tiny-3l@608	832 $\times$ 832	1088 $\times$ 1088	74.5	81.0	1.3
		832 $\times$ 832	85.4	92.6	1.6
		608 $\times$ 608	84.0	91.0	2.0
YOLOV4-tiny-3l@416	608 $\times$ 608	1088 $\times$ 1088	41.8	57.1	0.8
		832 $\times$ 832	64.4	71.4	1.0
		608 $\times$ 608	81.7	89.7	1.4
YOLOV4-tiny-3l@512	608 $\times$ 608	1088 $\times$ 1088	51.8	57.2	1.0
		832 $\times$ 832	74.6	81.1	1.3
		608 $\times$ 608	87.1	95.0	1.9
YOLOV4-tiny-3l@608	608 $\times$ 608	1088 $\times$ 1088	69.2	85.7	1.0
		832 $\times$ 832	83.8	91.9	1.4
		608 $\times$ 608	**87.4**	**95.1**	1.9
*Without tiling*					
YoloV4-tiny-3l@416	–	–	71.46	71.0	0.2
YoloV4-tiny-3l@512	–	–	79.4	78.0	0.2
YoloV4-tiny-3l@608	–	–	78.73	76.0	0.2

Bold values indicate the best-performing model

According to the experimental results, tiles with dimensions of 1088 $\times$ 1088, 832 $\times$ 832, and 608 $\times$ 608 provide the best detection performance for the proposed models. Considering the selected tile sizes and the proposed models’ network input resolution, which is 416 $\times$ 416, 512 $\times$ 512, and 608 $\times$ 608, the loss of detailed information due to resizing is reduced compared to directly resizing the full high resolution (4032 $\times$ 3024 pixels) image. As shown in Table 2, the YOLOV4-MOD model performs well on large tile sizes, but decreasing the tile size makes it difficult for the model to differentiate objects at a similar scale to *P. falciparum*, resulting in a large number of false-positive detections. However, lightweight models perform better on small size tiles as shown in Tables 3 and 4. The overlapping ratio between tiles was chosen based on experimental results on the validation dataset. The overlap between tiles prevents the model from missing objects due to image partitioning at tile boundaries.The proposed detection models achieved optimal detection accuracy with an overlap ratio of 0.2.

During inference time the tile size can be different from the one used during model training. Thus, in all experimental settings, the proposed detection models trained in one of the selected tile sizes were evaluated on three different tile sizes at inference time to investigate the effect of varying tile sizes at training and inference time. The proposed detection models perform better when the inference tile sizes are equal to or less than the training tile sizes. This could be due to CNN’s lack of strong generalization across scales [49] as well as the effect of input image downscaling to the network’s input resolution, which contributes to the loss of some detailed information. The YOLO4-MOD model’s detection performance does not improve when the network input resolution is increased. This could be due to the large size model’s deep network architecture, which contributes to equivalent localization features for objects on a similar scale to *P. falciparum*.

**Fig. 3:**
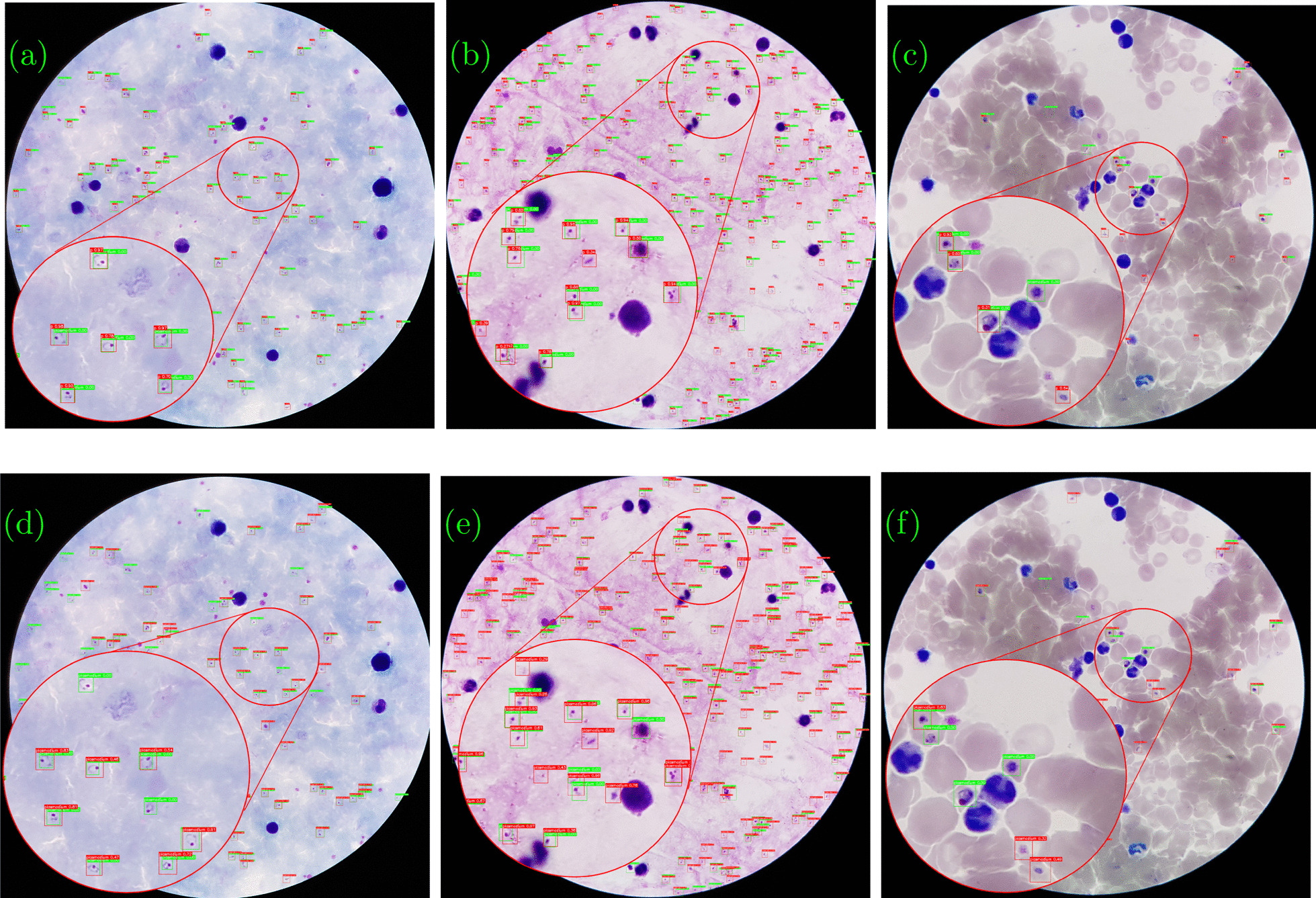
Sample visualization results of best-performing model (YOLOV4-tiny). The top row (**a**–**c**) shows detection results for three test images using the proposed method and the bottom row (**d**–**f**) shows detection results using the baseline approach. Ground truth bounding boxes are in green and predicted boxes are in red. The figure also shows how the images vary in color and infection rate

### Analysis of the experimental results

Tables 2, 3, and 4 show detailed experimental results for the various experimental settings described in "Experimental Setup" section. The experimental results show the effects of the various techniques used in this study, such as the proposed different detection networks, variation in network input resolution, and tile size variation both during the training and inference stages. Using the model development test data, which consists of 374 images from 30 patients, YOLOV4-tiny with 512 $\times$ 512 input resolution and tile size of 608 $\times$ 608 performs well both at train and inference time, with a maximum recall of 95.3% and a maximum average precision of 87.1%. On a similar test dataset, YOLOV4-tiny-3 l with 608 $\times$ 608 input resolution and tile size of 608 $\times$ 608 achieved a maximum recall of 95.1% and a maximum average precision of 87.4% at both train and inference time. Using a training tile size of 1088 $\times$ 1088 and an inference tile size of 832 $\times$ 832, the large size model (YOLOV4-MOD) with input resolution of 416 $\times$ 416 achieved a maximum recall of 95.1% and a maximum average precision of 85.5%. YOLOV4-tiny and YOLOV4-tiny-3 l outperform YOLOV4- MOD by 2.6 and 2 s, respectively, with better recall and average precision. Surprisingly, using the proposed tile-based approach, lightweight models achieve a better trade-off between detection performance and inference speed for *P. falciparum* detection than the large YOLOV4-MOD model. YOLOV4-tiny was chosen as the best lightweight model, with comparable performance to YOLOV4-tiny-3 l but a faster computation speed.

*Comparison to Baseline method* The performance of the proposed detection models was also compared to the respective baseline methods, which use high-resolution images directly during model training and inference. As shown in Tables 2, 3 and 4, the detection performance of models using the proposed tile-based approach is significantly better than their baseline counterparts. When compared to their baseline models, lightweight models performed 10 times slower in terms of computation speed, but their detection performance improved by significantly amount, with the yolov4-tiny-3 l model having an improvement in recall of about 17% and 38% for the yolov4-tiny model. The detection performance improvement due to the proposed approach for YOLOV4-tiny-3 l is lower than YOLOV4-tiny since the additional detection head enables these models to achieve better small object detection accuracy.

*Assessment of the selected model on external datasets* Furthermore, the performance of the chosen model (YOLOV4-tiny) was evaluated using two external datasets from a different domain. Using the entire dataset as test data, an average precision of 57.8% and recall of 75.1% was obtained by utilizing the first external dataset, which is a low-resolution image obtained from [10]. Similarly, using the second external data set obtained from [29], an average precision of 71.1% and recall of 86.3% were obtained.

In addition, the chosen model was fine-tuned and evaluated by dividing the two external datasets into training, validation, and testing groups and merging them with the model development dataset. As a result, on test data from the first external dataset, an average precision of 83.4% and recall of 94.7% were obtained, and on test data from the second external dataset, an average precision of 73.1% and recall of 96.3% were obtained.

### Qualitative results

Figure 3 depicts a qualitative comparison that demonstrates the profound effect of the tile-based approach on SOTA deep learning-based object detectors for *P. falciparum* detection from high-resolution thick smear microscopic images. As illustrated in the figure, detection models based on the proposed method produce very good detection results. The detection results in the first row of the sample visualization are based on the selected YOLOV4-tiny model and the proposed tile-based approach. The detection results for YOLOV4-tiny using the baseline approach are shown in the second row. Ground truth bounding boxes are green, while predicted bounding boxes are red. When comparing visualization results for image (a) and image (d), it is clear that the baseline approach produces more false positives (red boxes without green boxes) and false negatives (green boxes without red boxes). The same holds true for the images in columns 2 and 3. Even though the proposed method detects *P. falciparum* with high sensitivity in high-resolution input images, it still has a high number of false positives with low precision due to dark distractors with a very similar shape and color to the *P. falciparum* parasite. The research will be continued in the future to reduce the number of false positives by using hard negative mining techniques and adding a classifier head in front of the detection network.

**Fig. 4:**
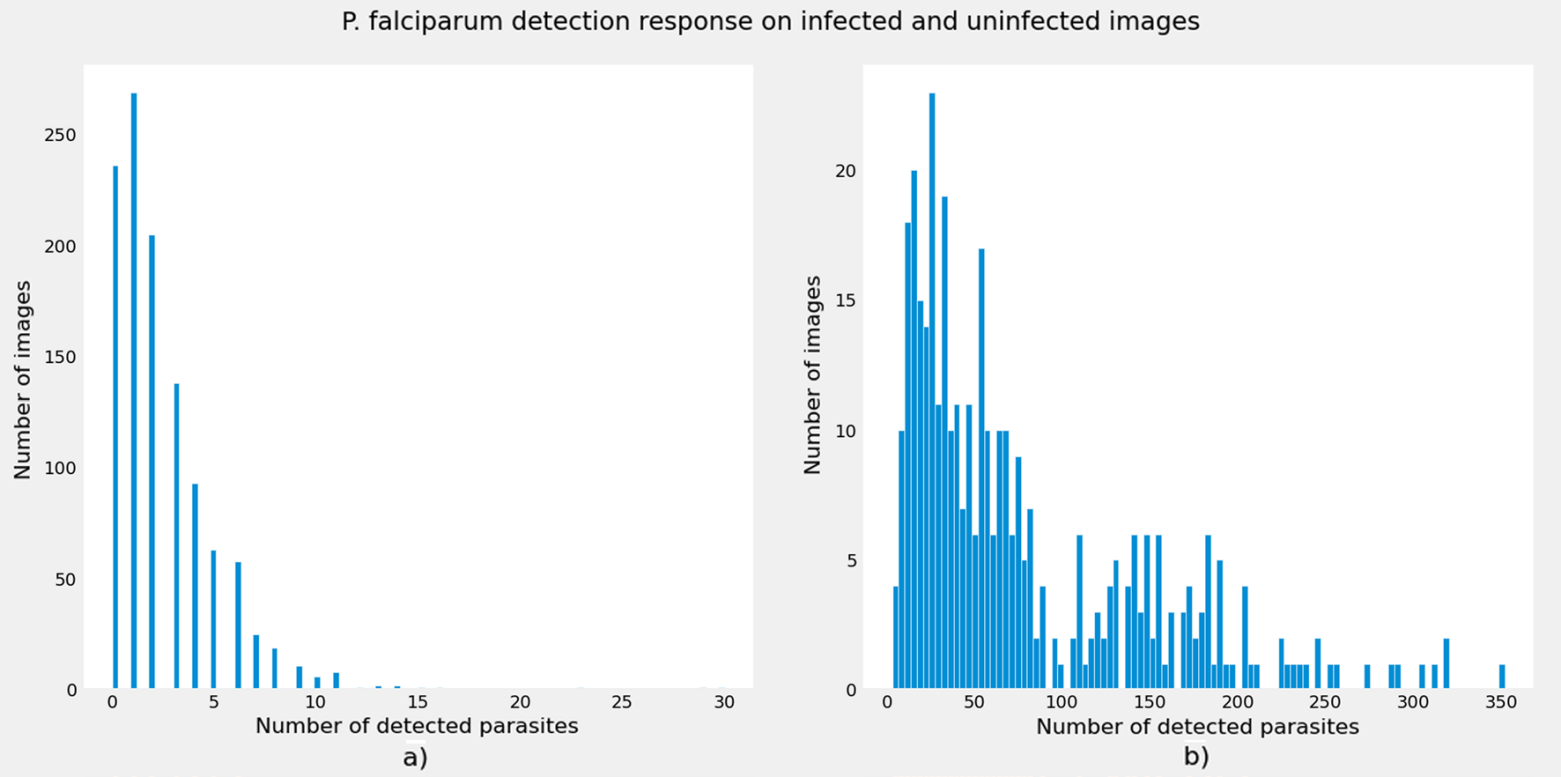
Detection response of the proposed method for **a** uninfected images and **b** infected images. From the histograms, it is evidenced that the proposed method is very effective in identifying *P. falciparum* on infected images and it is also effective on uninfected images with very few false positives due to distractors

## Discussion

The experimental results on the external datasets indicate that the proposed model struggles to generalize on a dataset collected in different environment. This is because the model development dataset was collected from one region (setting). The experimental results obtained by fine-tuning the selected model using external datasets indicate a significant increase in detection accuracy with improved generalization ability of the model. Therefore, this study demonstrates that training SOTA deep learning models with datasets collected from various health centers and geographic locations while considering real-time clinical procedures during manual microscopy diagnosis improved model generalization capability and detection accuracy.

The performance of the selected model utilizing the proposed tile-based approach was also compared with existing work [7], which uses a similar dataset to ours. In their work, parasite locations were pre-segmented using an intensity-based threshold technique and a custom CNN classifier on the segmented candidate regions for the final detection of *P. falciparum*. Compared to their work, the proposed method outperforms it by 7% in precision and 12% in recall. The detection performance results were compared to the two external datasets from different regions. For the external dataset obtained from [29], they obtained a precision of 67% and recall of 80%, which is significantly less compared to the proposed model’s detection performance, 73.1% average precision and 96.3% recall. For the other external dataset, which is obtained from [10] in their proposed model, they achieved high precision of 97%, but the recall is very bad at 22%, whereas by using the proposed method, an average precision of 83.4% and a recall of 94.7% was achieved.

Extensive validation experiments for the proposed method using datasets from various regions show that the proposed method can be used effectively for malaria parasite screening by recommending precise and suspicious *P. falciparum* parasite locations. This has the potential to significantly reduce the workload of laboratory technicians in malaria-endemic remote areas where there is a critical skill gap and a scarcity of experts. Surprisingly, when lightweight SOTA YOLOV4-based object detection models were compared to large-size complex models, the proposed method demonstrated a significant performance improvement. As a result, the proposed tile-based approach can even be used on low-end devices like smartphones, which can be integrated with the microscope without requiring a lot of computing power and memory.

To demonstrate the efficacy of the proposed approach for *P. falciparum* detection, detection performance the model on images with and without malarial parasite were analyzed. A total of 1141 images without malarial parasite obtained from 50 uninfected people were collected, and images without parasite from the model development test dataset were used. The proposed model generates one or two false positive detections in negative images due to artifacts that are suspicious due to their similarity to *P. falciparum* parasites and occurred as a result of staining and imaging procedures. Figure 4 shows that the best-performing model provides an effective detection response for images without parasite. Furthermore, the proposed tile-based image processing approach is applicable to other histopathological medical image analysis applications [50–52].

## Conclusion

Recent development of microscopy techniques accompanied by improvements in computer vision technologies hold enormous potential to aid medical diagnosis in developing countries where there is a critical shortage of resources. The challenges in manual microscopy for malaria parasite screening has motivated researchers to explore computer-aided diagnostic systems. Although the existing deep learning-based models for object detection in natural images has shown promising results, their application for a specific domain such as malaria parasite screening has its own challenges. The existing object detection models perform poorly on small object detection tasks such as malaria parasite detection. The other challenge is related to high-resolution microscopic images benefited from the advancement in digital data acquisition technologies such as high-resolution cameras. Directly applying high-resolution images by downscaling to SOTA detection network’s low resolution input degrades small object detection performance due to the detail information loss.

In this study, the performance of SOTA deep learning-based object detection models were increased for *P. falciparum* detection in high-resolution thick smear microscopic images. To achieve this, effectiveness of tile-based image processing which relies on training deep learning-based object detection models using tiles generated from an input high-resolution image was systematically evaluated. Besides, the proposed models were evaluated using datasets obtained from a different regions to validate their generalization ability and detection accuracy. Based on the extensive experimental analysis, lightweight YOLOV4 based models achieved a significant performance improvement using the proposed tile-based approach with 38% performance boost compared to its baseline method, while requiring only minimal additional computation cost. In addition, the proposed method outperforms detection results obtained by previous research works using similar datasets. The proposed malaria parasite screening technique has the potential to reduce workload of laboratory technicians by providing exact parasite locations or suspicious regions so that it can support the doctors to make their final decision. In the future work, we will focus on reducing the number of false-positive to improve precision of the proposed models by applying hard negative mining techniques and adding a classifier head that will be used to filter the detected objects.

## Data Availability

The datasets used are freely available online for research purpose. The model development dataset used in this study which was released by research work of [7] is available on this link https://data.lhncbc.nlm.nih.gov/public/Malaria/Thick_Smears_150/index.html (last accessed: 03/10/2022). The first external dataset used in this study released by research work of [10] is available here http://air.ug/downloads/plasmodium-phonecamera.zip (last accessed: 03/10/2022). The second external dataset used in this study released by research work of [29] is available on this link https://drive.google.com/drive/folders/1p45Dt-BJy8hhoI-rYnhcaL6IMl5FsFL-?usp=sharing (last accessed: 03/10/2022). The third external dataset for uninfected images released by research work of [20] is available on this link https://data.lhncbc.nlm.nih.gov/public/Malaria/NIH-NLM-ThickBloodSmearsU/NIH-NLM-ThickBloodSmearsU.zip (last accessed: 03/10/2022).

## Abbreviations

AP: Average precision
CAD: Computer-aided diagnostic
CNN: Convolutional neural network
FP: False positive
FN: False negative
GPU: Graphics processing unit
HR: High resolution
IOU: Intersection over union
NMS: Non-maximum suppression
PCR: Polymerise chain reaction
PRC: Precision-recall curve
RBCs: Red blood cells
RDT: Rapid diagnostic test
RGB: Red, green and blue
ROI: Region of interest
SOTA: State of the art
SSD: Single shot multibox detector
TP: True positive
WBC: White blood cell
WHO: World Health Organization
YOLOV4: You only look once version 4
